# Absorption of Radionuclides from the Fukushima Nuclear Accident by a Novel Algal Strain

**DOI:** 10.1371/journal.pone.0044200

**Published:** 2012-09-12

**Authors:** Hiroki Shimura, Katsuhiko Itoh, Atsushi Sugiyama, Sayaka Ichijo, Masashi Ichijo, Fumihiko Furuya, Yuji Nakamura, Ken Kitahara, Kazuhiko Kobayashi, Yasuhiro Yukawa, Tetsuro Kobayashi

**Affiliations:** 1 Department of Environmental Medicine, Interdisciplinary Graduate School of Medicine and Engineering, University of Yamanashi, Chuo, Yamanashi Japan; 2 Third Department of Internal Medicine, Interdisciplinary Graduate School of Medicine and Engineering, University of Yamanashi, Chuo, Yamanashi Japan; 3 Department of Business Development, Kitasato Academic Research Organization, The Kitasato Institute, Sagamihara, Kanagawa, Japan; 4 Department of Pharmacology, School of Medicine, Toho University, Ota-ku, Tokyo, Japan; 5 Department of Global Agricultural Sciences, Graduate School of Agricultural and Life Science, University of Tokyo, Bunkyo-ku, Tokyo, Japan; 6 Japan Biomass Corporation, Kashiwa, Chiba, Japan; University of Milano-Bicocca, Italy

## Abstract

Large quantities of radionuclides have leaked from the Fukushima Daiichi Nuclear Power Plant into the surrounding environment. Effective prevention of health hazards resulting from radiation exposure will require the development of efficient and economical methods for decontaminating radioactive wastewater and aquatic ecosystems. Here we describe the accumulation of water-soluble radionuclides released by nuclear reactors by a novel strain of alga. The newly discovered green microalgae, *Parachlorella* sp. *binos* (Binos) has a thick alginate-containing extracellular matrix and abundant chloroplasts. When this strain was cultured with radioiodine, a light-dependent uptake of radioiodine was observed. In dark conditions, radioiodine uptake was induced by addition of hydrogen superoxide. High-resolution secondary ion mass spectrometry (SIMS) showed a localization of accumulated iodine in the cytosol. This alga also exhibited highly efficient incorporation of the radioactive isotopes strontium and cesium in a light-independent manner. SIMS analysis showed that strontium was distributed in the extracellular matrix of Binos. Finally we also showed the ability of this strain to accumulate radioactive nuclides from water and soil samples collected from a heavily contaminated area in Fukushima. Our results demonstrate that Binos could be applied to the decontamination of iodine, strontium and cesium radioisotopes, which are most commonly encountered after nuclear reactor accidents.

## Introduction

Since the accidents at the Fukushima Daiichi Nuclear Power Plant on 11 March 2011, decontamination of the enormous quantities of water used to cool the reactors so that they can be shut down has become a critical issue. Indeed, it has been estimated that approximately 250,000 tones of water will be required by mid-January 2012 to cool down the reactors [1], injection of water at the rate of around half-a-million litres a day is required to keep the reactors at could shutdown [2]. In addition, the cost of the decontamination system developed by Areva (Paris, France) and Kurion (CA, US) may be as high as US $660 million, and the 2,000 cubic meters of the resulting radioactive sludge that will be generated will pose new long-term storage challenges by January, 2012 [1].

At the time of the Chernobyl nuclear accident, the plume that escaped from the reactor contaminated extensive areas as it was transported by the prevailing winds, not only in the vicinity of the reactor, but areas across the northern hemisphere [3], [4]. At the time, water-soluble radioactive nuclides, such as the radioisotopes of cesium (Cs), iodine (I), and strontium (Sr), contaminated aquatic ecosystems and food products [5]. Despite the relatively short half-life of radioiodine, the incidence of thyroid cancer in children living in radioiodine-contaminated areas increased significantly [3]. Subsequent phases of environmental contamination were primarily due to ^137^Cs, ^134^Cs, and ^90^Sr, which was considered harmful as Cs and Sr are concentrated in human muscle and bone, respectively [3]. At the Fukushima Daiichi Nuclear Power Plant, 18 PBq of ^134^Cs, 15 PBq of ^137^Cs, 2.0 PBq of ^89^Sr, 0.14 PBq of ^90^Sr, and 160 PBq of ^131^I have been released into the environment to date [6], and radionuclides from the reactors in Fukushima have already been detected in Russia and Greece [7]. Thus, developing an effective and economical method for removing these radionuclides from radioactive wastewater and aquatic ecosystems has become an increasingly important issue.

The Fukushima nuclear accident was triggered by an act of God in the form a megathrust earthquake and associated tsunami. Since the natural environment exhibits an innate tendency towards recovery [8], we attempted to identify at least one of these recovery mechanisms that could be applied to the decontamination of radionuclides from the environment, rather than investigating artificial methods of decontamination. In this report, we show that a newly discovered green microalga efficiently absorb radioactive isotopes of iodine, strontium, and cesium.

## Results

**Figure 1:**
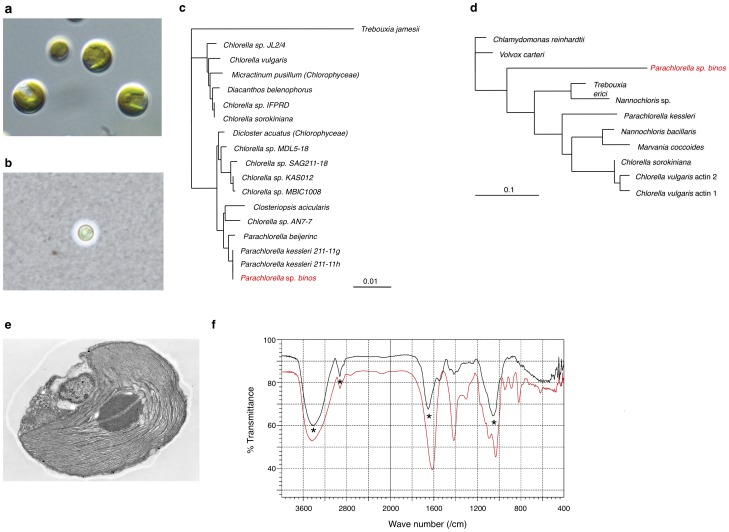
Identification of a novel strain of green algae, *Parachlorella* sp binos (Binos). **a**, Microscopic image of *P.* sp *binos* (Binos) (x2000). **b**, India ink staining of *P.* sp *binos* (Binos) (x400). India ink was diluted with water to make a 10% solution. **c, d**, Phylogenetic analysis of members of the phylum Chlorophyta based on (**c**) 18S rRNA gene and (**d**) actin amino acid sequences. **e**, Transmission electron micrographs of *P.* sp *binos* (Binos). **f**, Infrared spectra of the extracellular matrix of *P.* sp *binos* (red line) and purified sodium alginate (standard; black line) analyzed by Fourier transform infrared spectroscopy from 400 to 4000 cm^−1^.

On the morning after a recent typhoon, we found floc of green algae in activated sludge at a wastewater treatment plant in Kitaibaraki, Ibaraki, Japan, approximately 78 km from the Fukushima Daiichi Nuclear Power Plant [9]. We isolated a strain of green microalgae that was viable at high temperatures up to 60°C and was resistant to acidic and alkaline conditions at a pH range of 3–11 (data not shown). This alga was also capable of growing in fresh water and seawater. This strain was unique in that it had abundant chloroplasts (Figure 1A) and an extracellular matrix that was observed by India ink staining under a light microscope (Figure 1b). To clarify the taxonomic affiliation of this strain within the phylum Chlorophyta, we sequenced part of the 18S rRNA and an actin gene as described previously [10]. Phylogenetic analysis of the 18S rRNA gene sequences showed that the novel algal strain was embedded within a clade containing the members of the class Trebouxiophyceae and shared a node with *Parachlorella kessleri* (Figure 1c). However, amino acid sequences encoded by the actin mRNA revealed that the newly isolated algal strain appeared to be less closely related and *P. kessleri* (Figure 1d). Taken together, these findings suggested that the novel algal strain could be assigned to the genus *Parachlorella*, and we designated the strain *Parachlorella* sp. *binos* (Binos).

Transmission electron microscopy revealed that most of the cytosol of the alga was occupied by chloroplasts (Figure 1e), whereas the chloroplasts in a *Chlorella* sp. that is considered to be closely related to Binos, are restricted to the margins of the cytosol [11]. The external surface of the novel alga was also covered by a thick extracellular matrix (Figure 1e), which is not found in the *Chlorella* sp. [11]. Fourier transform infrared spectroscopy revealed that the extracellular matrix mainly consisted of alginate, an anionic polysaccharide commonly found in the cell walls of brown algae, but not in green algae [8] (Figure 1f).

**Figure 2:**
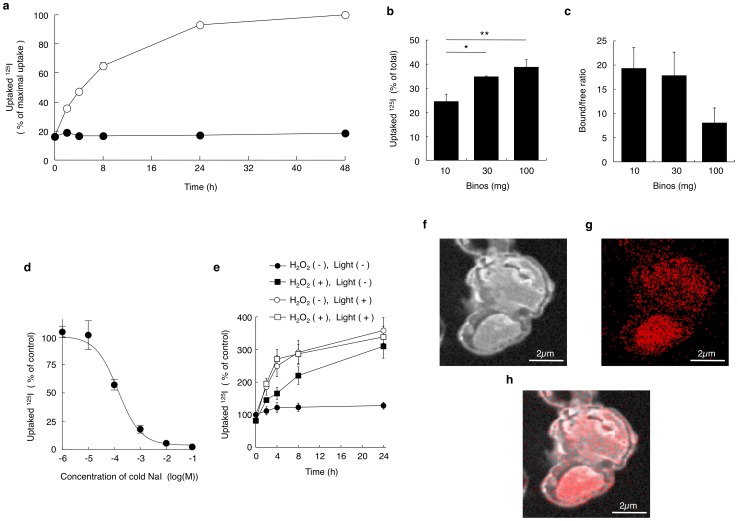
Radioiodine uptake by *P.* sp *binos* (Binos). **a**, Ten mg/ml of *P.* sp *binos* was incubated with 1.48 kBq of Na^125^I at room temperature under illumination at 2000 lux (open circles) or in the dark (solid circles) (n = 4). Radioactivity accumulated in algal cells were measured, and data are shown as maximal uptake. **b**, Algal cells incubated with Na^125^I at room temperature under illumination for 24 h. Radioactivity accumulated in algal cells were measured, and data are shown as % of total radioactivity. *, *P*<0.05, **, *P*<0.01 (n = 4). **c**, After 24 h incubation, pellet and supernatant radioactivities were measured and the bound/free ratios of ^125^I were calculated (n = 4). **d**, Ten mg/ml of wet algal cells were incubated with nonradioactive NaI and 1.48 kBq of Na^125^I (n = 4). Radioactivity accumulated in algal cells were measured, and data are shown as % of control uptake without nonradioactive NaI **e**, Algal cells were incubated with or without 10 µM of hydrogen peroxide (H_2_O_2_) for 24 h (n = 3). **f, g**, After incubation of algal cells with 1 µM of sodium iodide for 24 h, elemental distribution of ^12^C^14^N (f) and ^127^I (g) on the section of algal cells were visualized by NanoSIMS. **h**, Marged image of Figure 2f and 2g.

When cultured under illumination (2000 lux), absorption of radioiodine by the alga was observed two hours after the addition of Na^125^I, and maximum uptake of ^125^I was observed after 24 to 48 h (Figure 2a). This ability of Binos to adsorb iodine was found to be light-dependent as algal cells cultured in the dark did not take up radioiodine. In addition, ^125^I uptake was also correlated with the density of algal cells (Figure 2b); 100 mg/ml of cells in 1 ml of H_2_O with Na^125^I accumulated 38.7±3.1% of total radioiodine after 24 h. The bound/free ratio of ^125^I calculated with the formula described in *Materials and Methods* reached 19.3±0.5 when 10 mg/ml of wet Binos added to the reaction (Figure 2c). Dehydration of Binos increased the bound/free ratio of ^125^I up to 386±62. A radioiodine binding study using nonradioactive iodine as a competitor revealed that the dissociation constant (*K_d_*) and the maximal binding capacity (B_max_) of iodine to algal cells were 141 µM (95% confidence interval (CI); 104–191 µM) and 2.52 nmol/mg wet weight (CI: 2.33–2.71 nmol/mg wet weight; 50.4 nmol/mg dry weight), respectively.

The process of photosynthesis is known to produce superoxides, such as hydrogen peroxide [12], and haloperoxidases, both of which mediate halide oxidation by hydrogen peroxide and have been implicated in iodine uptake by marine brown algae such as *Laminaria* sp [13], [14]. We found that the addition of 10 µM of H_2_O_2_ stimulated radioiodine uptake in dark conditions in a time-dependent manner (Figure 2e). This finding suggested that iodine uptake by Binos was, at least in part, mediated by the increase in the oxidative power associated with photosynthesis.

In order to analyze the distribution of accumulated iodine ions at the subcellular scale, high-resolution secondary ion mass spectrometry (SIMS) was performed. Distribution of stable isotopes of carbon and nitrogen, ^12^C and ^14^N, localized the algal cells and those extracellular matrix (Figure 2f). After 24h incubation with 1 µM of ^127^I under the light, accumulated iodine was mainly localized in cytosol (Figure 2g, h). This result clearly indicated that radioiodine ions are also transported across the cell wall and membrane.

**Figure 3:**
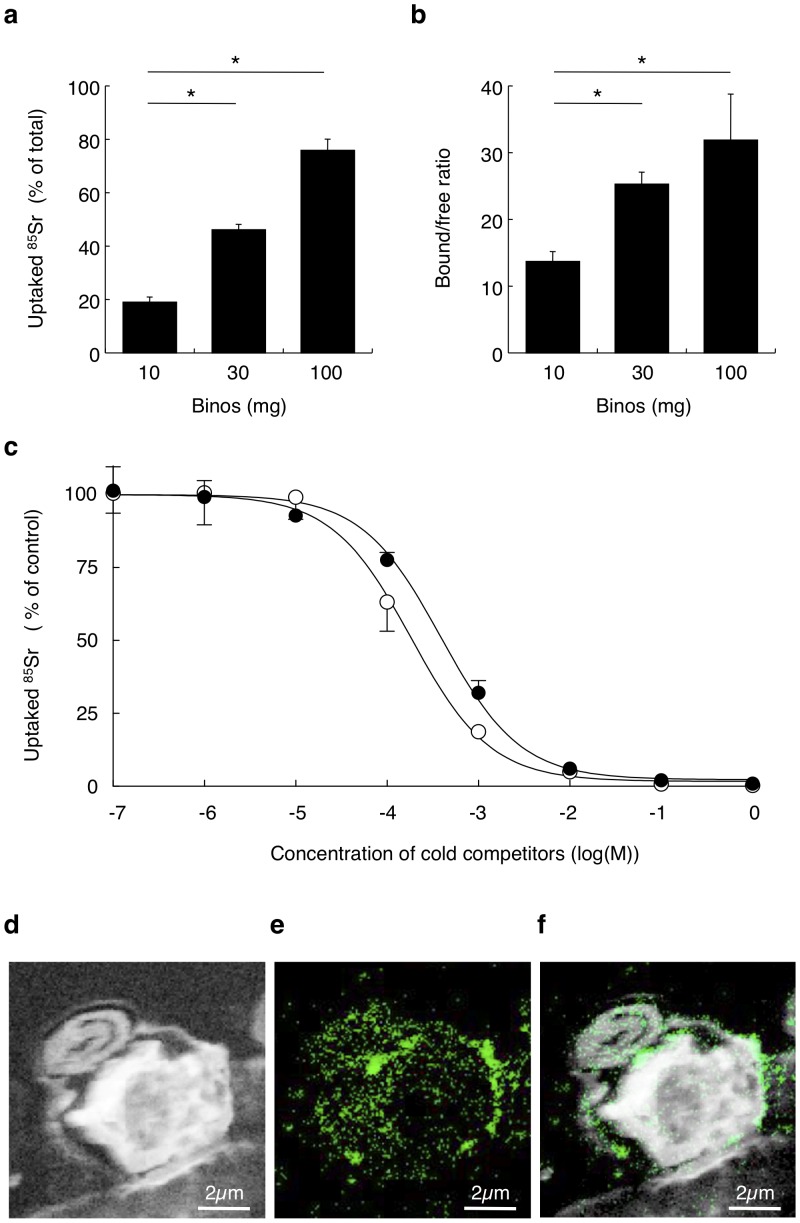
Binding of radioisotopes of strontium with *P.* sp *binos.* **a**, 10, 30, and 100 mg/ml of wet algal cells were incubated with 2.17 kBq of ^85^SrCl_2_ at room temperature for 10 min. Radioactivity accumulated in algal cells were measured, and data are shown as % of total radioactivity. *, *P*<0.0001 (n = 6). **b**, After 10 min incubation, pellet and supernatant radioactivities were measured and the bound/free ratios were calculated. *, *P*<0.0001 (n = 6). **c**, Ten mg/ml of wet algal cells were incubated with nonradioactive SrCl_2_ (solid circles) or CaCl_2_ (open circles) and 2.17 kBq of ^85^SrCl_2_ at room temperature for 10 min (n = 4). Radioactivity accumulated in algal cells were measured, and data are shown as % of control uptake without nonradioactive SrCl_2_. **d**, **e,** After incubation of algal cells with 1 µM of strontium chloride, elemental distribution of ^12^C^14^N (d) and ^88^Sr^35^Cl (e) on the section of algal cells were visualized by NanoSIMS. **e,** Marged image of Figure 3d and 3e.

The alga also exhibited an ability to bind ^85^Sr, a γ-ray emitting isotope, in a density-dependent manner; for example, at densities of 100 mg/ml, the alga accumulated 75.9±4.2% of the total ^85^Sr added (Figure 3a). For 100 mg of wet alga, the bound/free ratio of ^85^Sr was 31.9±6.7 (Figure 3b), and that in dried alga was 638±134. Binding of ^85^Sr, which was observed after 1 min and reached maximal levels after 10 min, occurred independently of exposure to light (data not shown). An ^85^Sr-binding study using nonradioactive Sr as a competitor revealed that the *K_d_* and B_max_ associated with Sr binding to algal cells were 387 µM (CI: 318–472 µM) and 15.5 nmol/mg (CI:14.9–16.1 nmol/mg; wet weight), respectively (Figure 3c). When the alga was dehydrated, B_max_ was 310 nmol/mg (CI: 298–322). Competition analysis showed that the *Ki* associated with the binding of Ca to the alga was 183 µM (141–238 µM), which was significantly lower than the *K_d_* observed with Sr, even though both elements are divalent ions. Alginate in the extracellular matrix is an anionic polysaccharide with carboxyl groups that electrostatically cross-link with divalent cations, such as Ca or Sr. It may thus be possible to exploit the thick extracellular matrix that is characteristic of the alga to accumulate radioactive cations. SIMS mapping of the nonradioactive isotope of strontium clearly showed localized distribution of Sr at the outer surface of extracellular matrix (Figure 3d–f), while only a little of Sr were detected in the cytosol.

**Figure 4:**
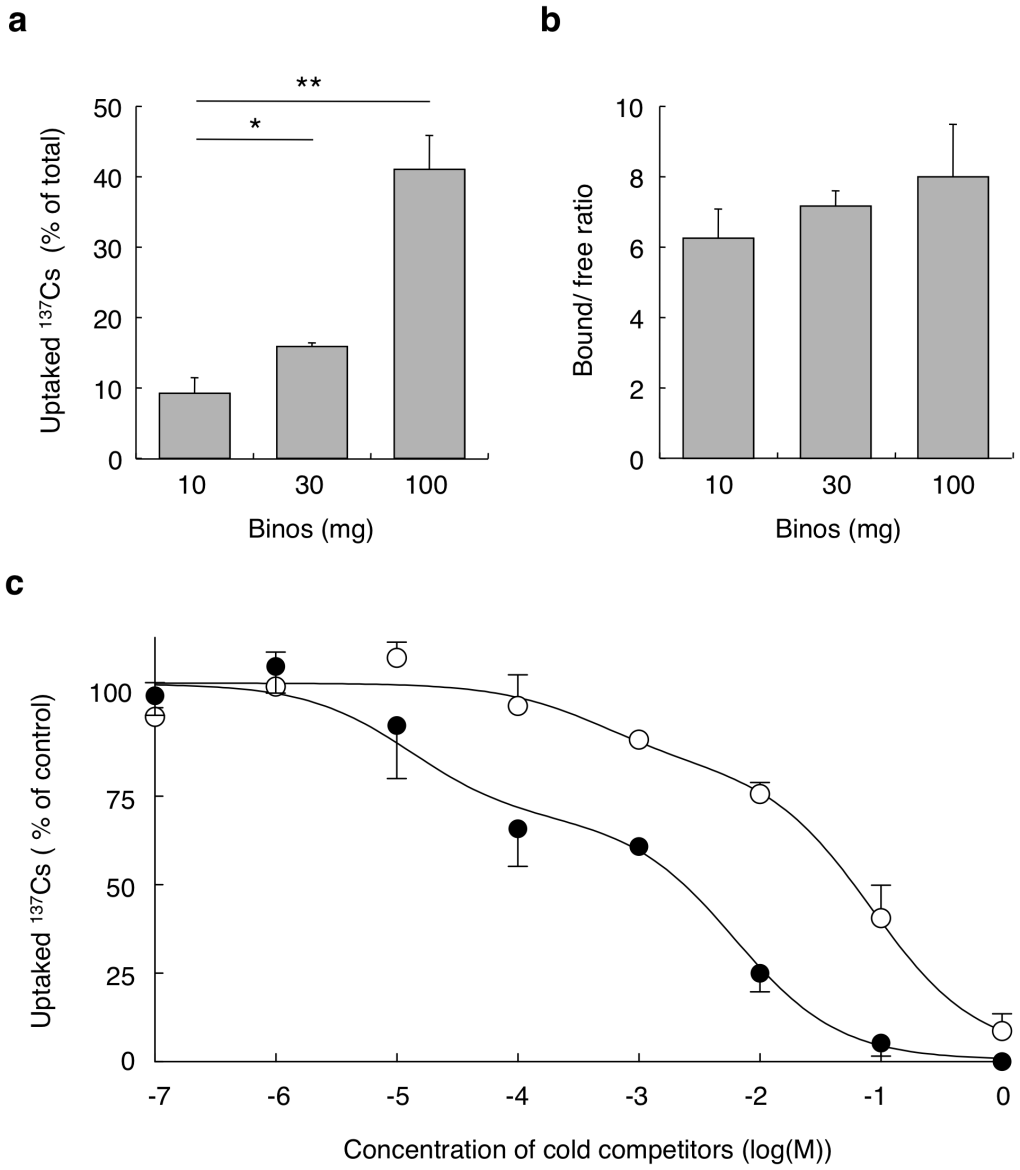
Binding of radioisotopes of cesium with *P.* sp *binos.* **a**, 10, 30, and 100 mg/ml of wet algal cells were incubated with 1.92 kBq of ^137^CsCl at room temperature for 10 min. Radioactivity accumulated in algal cells were measured, and data are shown as % of total radioactivity (mean ± SD). *, *P*<0.05, **, *P*<0.01 (n = 4). **b**, After 10 min incubation, the radioactivities of pellets and supernatant were measured and the bound/free ratios were calculated (n = 4). **c**, Ten mg/ml of wet algal cells were incubated with nonradioactive CsCl (solid circles) or NaCl (open circles) and 1.92 kBq of ^137^CsCl at room temperature for 10 min. Radioactivity accumulated in algal cells were measured, and data are shown as % of control uptake without nonradioactive CsCl. Non-linear curve fitting to the model of two binding sites with high and low affinities was performed using the CsCl and NaCl competition data (n = 4).

We then analyzed the capacity of the alga to adsorb and accumulate radioactive Cs, which has prompted extensive concern in Japan due to reports of food and soil contamination in recent weeks. The alga accumulated up to 41.0±4.8% of the ^137^Cs 10 min after addition (Figure 4a). When 100 mg/ml of Binos was added, the bound/free ratio of ^137^Cs was 8.00±1.49 (wet weight) and 160±30 (dry weight) (Figure 4b). Binding of ^137^Cs to the alga was observed after 1 min, with maximal levels of binding observed after 10 min in the absence of illumination (data not shown). Competition analysis using nonradioactive Cs revealed that Cs bound to two sites on the alga with different affinities; *K_d_* values for the high and low affinity binding sites were 19.90 µM (CI: 6.20–64.2 µM) and 7.25 mM (CI: 3.11–16.90 mM), respectively (Figure 4c). The B_max_ of the high and low affinity binding sites were 21.3 (CI: 16.6–25.9) pmol/mg wet weight (426 pmol/mg dry weight), and 7.80 (CI: 6.43–9.17) nmol/mg wet weight (156 nmol/mg dry weight), respectively. We also examined the binding affinity of sodium ions by competition analysis, as sodium has similar characteristics to Cs ions and contamination of radioactive sodium chloride was reported in wastewater at the Fukushima site [1]. Sodium also bound to the two binding sites, and *Ki* values of 0.585 mM (CI: 0.052–6.64) and 92.1 mM (CI: 36.9–230.4) were obtained (Figure 4c). Compared to Cs, the lower binding affinity of sodium ions could potentially be applied to the decontamination of ^137^Cs from sodium chloride-contaminated water.

**Figure 5:**
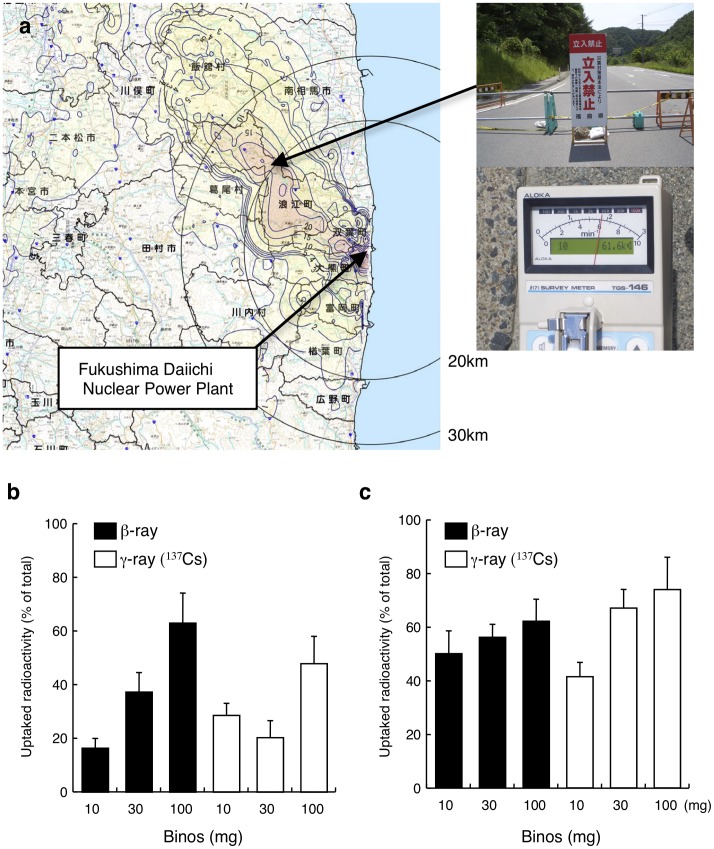
Binding of radionuclides in contaminated water and soil samples from Fukushima. **a**, Soil and water soil samples were collected from Namie-machi, approximately 20 km from the Fukushima Daiichi Nuclear Plant. The upper photograph shows a signboard prohibiting entry to the 20 km exclusion zone. Gross radioactivity of ground samples measured using a Geiger-Müller counter was approximately 60 kcpm (lower photograph). Water samples (**b**) and soil-water extracts (**c**) contaminated by radioactive fallout were incubated with algal cells under illumination at 2000 lux for 8 h. Gross β radioactivity (closed bars) and γ radioactivity (open bars) from ^137^Cs were measured by liquid scintillation and γ counters, respectively.

We collected soil and water samples that had been contaminated by radioactive fallout from Namie-machi, Fukushima (37°32′30.85″N, 140°51′40.75″E), a hot spot located 20 km from the nuclear plant. Average ground radioactivity measurements in the area were approximately 60 kcpm (Figure 5a). Recent reports have shown that soil deposition of ^134^Cs, ^137^Cs, ^89^Sr, and ^90^Sr in this area was 1.94×10^3^, 2.21×10^3^, 1.60×10^4^, and 3.7×10^3^ kBq/m^2^, respectively [15], [16]_ENREF_14. ^137^Cs detected in water samples and extracts from soil samples were approximately 85 and 79 Bq/ml, respectively, which is compatible with the reported ^137^Cs radioactivity in this area, 2.21×10^3^ kBq/m^2^ ( = 221 Bq/cm^2^). After incubation of algae with water samples or extracts from soil samples for 8 h, radioactivity from ^137^Cs or gross β radioactivity in the pellet constituted by algae was quantified. 100 mg/ml of algal cells accumulated 63.0% of gross β radioactivity by incubation in water samples. Up to 47.9% of ^137^Cs (γ-ray) in water samples was also accumulated by 100 mg/ml of Binos (Figure 5b). Similarly, in soil-water preparations, the alga accumulated both β-ray emitting radionuclides and ^137^Cs by as much as 62.2% and 74.0%, respectively (Figure 5c).

## Discussion

Here we report the characteristics of newly discovered green microalga, *Parachlorella* sp. *binos* (Binos), which has thick alginate-containing extracellular matrix and abundant chloroplasts. Binos exhibited light-dependent uptake of radioiodine and highly efficient incorporation of the radioactive isotopes strontium and cesium in a light-independent manner. In addition, we show accumulating radioactive nuclides by Binos from water and soil samples collected from a heavily contaminated area in Fukushima.

Brown algal species, such as the kelp, *Laminaria digitata*, have widely been considered to be the most effective living accumulators of iodine, and tissue concentrations exceeding 50 mM have been reported [14]. In addition, marine brown algae have also been reported to absorb divalent or trivalent heavy metal ions, and B_max_ values of approximately 120–2030 mM have been obtained [8]. Our findings show that Binos was capable of accumulating 50.4, 310, and 156 mM iodine, Sr, and Cs, respectively. Indeed, dried preparations of this algal strain exhibited absorption efficiencies that were comparable with those of brown algae. It was recently reported that *Closterium moniliferum*, a desmid green alga, accumulated Sr [17]. In this study, we report that this novel green algal strain, Binos, was capable of simultaneously adsorbing the most abundant radioisotopes released by the reactors at the Fukushima Daiichi Nuclear Power Plant. Development of decontamination systems that are both efficient and economical are urgently required in order to remediate areas affected by this accident and to reduce health risks resulting from radiation exposure.

Transmission electron microscopy of Binos revealed its unique characteristics, a thick alginate-containing extracellular matrix and abundant chloroplasts, both of which were not observed in *Parachlorella kessleri* [11]. Our study showed that radioiodine was accumulated into the cytosol under light exposure or in the presence of hydrogen peroxide. These findings suggest that iodine uptake by Binos was, at least in part, mediated by the increase in the oxidative power associated with photosynthesis. It is therefore possible that the abundance of chloroplasts in this algal strain can account for the efficient accumulation of radioiodine. In contrast to iodine, SIMS mapping of strontium clearly showed localized distribution of Sr at the outer surface of extracellular matrix. It may thus be possible to exploit the thick extracellular matrix to accumulate radioactive cations.

In the radionuclide uptake assay, we calculated the bound/free ratio that is important for estimation of decontamination efficiency. As shown in Figure 4b, the bound/free ratio of ^137^Cs did not differ between the three groups with different amount of alga. This result suggests that the amount of cesium accumulated in algae is dependent on the constant bound/free ratio, and that the binding reaction with cesium might be reversible reaction. In contrast, the bound/free ratio of ^85^Sr was increased paralleled with the amount of alga (Figure 3b). This result might be reflected by the higher affinity in strontium binding in comparison with that in cesium binding. Although the Bmax in cesium binding to low affinity binding site, 7.8 nmol/mg wet Binos (Figure 4c), was comparable with that in strontium binding, 15.5 nmol/mg wet Binos (Figure 3c). Affinity of strontium binding, *Kd* = 387 µM, was significantly higher in comparison with that of cesium binding to lower affinity binding site, *Kd* = 7.25 mM. In addition, the fact that a fraction of strontium was identified in the algal cytosol (Figure 3d–f) suggests that the binding reaction with strontium might include somewhat of irreversible incorporation into the cell. In the case of ^125^I, the bound/free ratio was in inverse proportion to the amount of algae (Figure 2c). When 10 mg of algae were incubated in the culture wells, light was supposed to expose to all algae. However, in such condition as incubation with much amount (100 mg) of algae, which were overlaid with each other in the incubation wells, mutual inhibition of light exposure to algae might decline the efficiency of uptake with decreased bound/free ratio. Therefore, we suggest that the true uptake efficiency might be shown in the results using 10 mg/ml of algae.

We collected soil and water samples in Namie-machi, Fukushima, where one of hottest spots located in the northwest of Fukushima Daiichi Nuclear Power Plant. We show that Binos accumulated ^137^Cs and β-ray emitting radionuclides in the water and soil samples. In comparison with the water samples shown in Figure 5b, Binos accumulated ^137^Cs and β-ray emitting radionuclides more effectively in extracts from soil samples (Figure 5c). Soil and visible macro-debris were completely removed from extracts by three-times centrifugation. Therefore, it is unlikely that some soil components unbound to Binos settled down into pellets after incubation with Binos. We speculated that positively charged unvisible micro-debris containing radionuclides derived from soil might bind to negatively charged alginate in the extracellular matrix of Binos.

Zeolite is an aluminosilicate that also efficiently absorbs radioactine iodine, cesium, and strontium [18], and has been utilized in the water decontaminating system in the Fukushima Daiichi Nuclear Power Plant. However, the volume of zeolite is not able to be reduced furthermore. In this report, we demonstrate that this novel alga strain can simultaneously decontaminate all predominant radioisotopes released from Fukushima Daiichi nuclear power reactors, and that this strain can reduce the volume of radioactive wastes at the ratio of 1∶160–638 with the drying step. In addition, advantage to use this strain rather than any strains of natural seaweeds is that this strain can rapidly proliferate in industrial plants [9] and can be applied to various styles of decontamination systems because of its microscopic size. Since contaminated water in Fukushima Daiichi Nuclear Power Plant is expected to be in various conditions such as high concentration of salts, high temperature, low, or high pH, the characteristics of this strain that is resistant to hot, high salt, acidic, or alkaline condition must be another advantage. Development of decontamination system, which is expected to be efficient and economical, is urgent assignment to settle the accident and reduce risk of health hazard.

## Materials and Methods

### Sampling and Cultures of Algal Strains

We isolated a strain of green microalgae from activated sludge at a wastewater treatment plant in Kitaibaraki, Ibaraki, Japan, approximately 78 km from the Fukushima Daiichi Nuclear Power Plant. No specific permits were required for this studies and obtaining algal species. No specific permissions were required for the sampling location. We confirm that the location is not privately-owned or protected in any way, and that the field studies did not involve endangered or protected species. Algae were isolated from activated sludge by treatment with 0.1% povidone iodine. We isolated a strain of green microalgae that was viable at high temperatures up to 60°C and was resistant to acidic and alkaline conditions at a pH range of 3–11. The alga was cultured in inorganic growing medium containing 111 mM glucose, 2.5 mM KNO_3_, 0.3 mM MgSO_4_, 1.3 mM KH_2_PO_4_, 0.43 mM NaCl, 0.23 mM CaCl_2_, 1.7 mM NH_4_H_2_PO_4_, 0.14 mM EDTA, 47 nM FeCl_3_, 93 µM H_3_BO_4_, 18 µM MnCl_2_, 1.5 µM ZnSO_4_, 0.63 µM CuSO_4_, and 24 nM (NH_4_)_5_Mo_7_O_31_ under illumination (13000–18000 lux) and an atmosphere of 15% CO_2_ at 30°C^6^. The algal strain was deposited at The National Institute of Advanced Industrial Science and Technology (Tokyo, Japan) under accession number FERM BP-10969.

### Amplification of 18S rRNA Gene Sequences

Total DNA was extracted using an ISOPLANT kit (Nippon Gene, Tokyo, Japan) according to the manufacturer’s instructions. 0.1 µg of purified DNA was used as a template for PCR, which was performed using the forward primer 5′-GTAGTCATATGCTTGTCTC-3′ and the reverse primer 5′-GGCTGCTGGCACCAGACTTGC-3′, with 30 cycles of 1 min at 94°C, 1 min at 55°C and 1 min for 72°C. Amplified PCR products were then ligated into p-GemT vectors (Promega, Madison, WI) and sequenced using an ABI3700 automated DNA sequencer (Life Technologies, Carlsbad, CA).

### Amplification of Actin cDNA

Total RNA was extracted by phenol/chloroform precipitation after treatment of the algal cells with Proteinase K and DNase. mRNA was then reverse transcribed using a PrimeScript kit/PrimeScript reverse transcriptase (Takara Bio, Otsu, Shiga, Japan) and the PCR was performed using cDNA as a template with the forward primer 5′-ATGACSCAGATCATGTTYGAGAC -3′ and the reverse primer 5′-CCACATYTGCTGGAAGGTGG-3′ for 30 cycles of 1 min at 94°C, 1 min at 50°C and 1 min at 72°C. Actin cDNA sequences were determined as described above.

### Phylogenetic Analysis

The 18S rRNA and actin cDNA were amplified by reverse transcription-PCR as described above. rRNA nucleotide and actin amino acid sequences for the phylum Chlorophyta were obtained from the GeneBank database and were aligned using the MEGA5 software package [19].

### Fourier Transform Infrared Spectroscopy

The extracellular matrix of the algal cells was precipitated by treating the supernatant of the incubation medium with ethanol Then, either the precipitated sample or purified sodium alginate (Nacalai Tesque, Kyoto, Japan) was analyzed using the attenuated-total-reflection technique with an IRPrestige-21 Fourier Transform Infrared Spectroscopy (Shimadzu Corporation, Kyoto, Japan).

### Radionuclide Uptake Assay

*Parachlorella* sp *binos* (Binos) was grown at 30°C for 72 h in an growing culture medium containing 111 mM glucose, 50 mM KNO_3_, 10 mM MgSO_4_, 9.2 mM KH_2_PO_4_, 0.57 mM K_2_HPO_4_, 31 mM NaCl, 0.11 mM FeSO_4_, 0.14 mM EDTA, 46 µM H_3_BO_4_, 9 µM MnCl_2_, 0.8 µM ZnSO_4_, 0.3 µM CuSO_4_, and 12 nM (NH_4_)_5_Mo_7_O_31_. The grown algae was further incubated at 30°C in inorganic culture medium containing 2.5 mM KNO_3_, 0.3 mM MgSO_4_, 1.3 mM KH_2_PO_4_, 0.43 mM NaCl, 0.23 mM CaCl_2_, 1.7 mM NH_4_H_2_PO_4_, 47 nM FeCl_3_, 93 µM H_3_BO_4_, 18 µM MnCl_2_, 1.5 µM ZnSO_4_, 0.63 µM CuSO_4_, and 24 nM (NH_4_)_5_Mo_7_O_31_. After 16 h incubation, the medium was centrifuged at 300×g and the pellet was resuspended in water and incubated at room temperature for 1 h. The algal pellet was then centrifuged at 300×g for 10 min and resuspended in H_2_O. For the uptake assay, 10, 30, or 100 mg/ml of the harvested alga were incubated with 1.48 kBq of Na^125^I (15.7 pM), 2.17 kBq of ^85^SrCl_2_ (26 nM), or 1.92 kBq of ^137^CsCl (2.91 µM) in 24 well plates. After incubation with radioisotopes, the algae were centrifuged at 3000×g for 10 min at 4°C and the weight and radioactivity of the pellet was determined after removal of the supernatant. Unless stated otherwise, data were presented as means ± SD.

The bound/free ratio was calculated with the following formula.
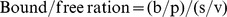
where b is radioactivity accumulated by Binos (cpm), p is weight of the pellet (g), s is radioactivity of the supernatant (cpm), and v is volume of the supernatant (ml).

For the competition assay, the algal strain was simultaneously incubated with radioactive isotopes and nonradioactive NaI, SrCl2 or CsCl in 1 ml of H_2_O. Non-linear curve fitting and calculation of B_max_, *K_d_*, IC_50_, and *Ki* was then performed using GraphPad Prism software (version 5.0d for Macintosh, GraphPad Software, San Diego, CA), and its statistical significance was analyzed with a t-test.

### Secondary Ion Mass Spectroscopy (SIMS) Imaging

SIMS examinations were carried out on a NanoSIMS-50 Ion Microprobe (CAMECA, Courbevoie, France) [20]. To analyze the intracellular distribution of iodine, algal cells (10 mg/ml) were incubated with 1 µM of sodium iodide for 24 h. For imaging of strontium, algal cells were incubated with 1 µM of strontium chloride for 1 h. After centrifugation, the samples have been deposited on papers foils. The papers foils have been projected on copper block at N_2_ temperature (−180°C). After that, the samples have been dehydrated by sublimation of the ice before being plunged in cooled (−30°C) Unicryl resin. Resin infiltration has been carried out 24 h at –30°C. Afterwards UV polymerization was achieved 24 h at –30°C and 48 h by rising temperature to room temperature. The obtained resin blocs were sectioned by ultra microtome (400 nm) and deposited on NanoSIMS50 silicon wafer. SIMS observations were carried out on a NanoSIMS 50 micro beam analyzer using a Cs+ primary source. Distribution of negative ions: ^12^C^14^N^−^, ^88^Sr^35^Cl^−^, and ^127^I^−^ were recorded as a matrix of 256 × 256 image points.

### Contaminated Water and Soil Sampling and Binding Assay

Water and soil samples were collected on 15 June 2011 in Namie-machi, an area that had been contaminated by radiation fallout from the Fukushima Daiichi Nuclear Power Plant 20 km away. Water samples were corrected from a small pool, in which water did not flow into or out. Soil samples were corrected at the shoulder of the road. At the time of collection, the radiation dose at this location was reported to be ≥20 µSV/h [21]. Radioactive nuclides from soil samples were extracted by incubating the samples at 80°C with equal quantities of H_2_O overnight [22]. Centrifugation of the water samples and soil-water preparations were performed three times at 3000 ×g for 10 min to remove any soil or debris. The supernatant was then incubated with the Binos alga for 8 h, after which the samples were centrifuged at 3000×g for 10 min at 4°C. Radioactivity from ^137^Cs in the pellet constituted by Binos was quantified by measurement of γ-ray in the energy region between 547 keV and 770 keV. Gross β radioactivity in the energy region between 500 keV and 2000 keV where β-ray from ^137^Cs, ^89^Sr, and ^90^Sr were included were measured by a liquid scintillation counter.
