# Correction: Absorption of Radionuclides from the Fukushima Nuclear Accident by a Novel Algal Strain

**DOI:** 10.1371/journal.pone.0095903

**Published:** 2014-04-16

**Authors:** The PLOS ONE Staff

**Notice of Republication**

This article was republished on March 28, 2014, due to the release of confidential or copyrighted information. Please download the article again to view the corrected version.
